# A novel prognosis-prediction model based on coagulation indicators in secondary hemophagocytic lymphohistiocytosis

**DOI:** 10.1007/s00277-023-05398-w

**Published:** 2023-08-10

**Authors:** Shixuan Wang, Kebing Lv, Yulan Zhou, Xiaoye Cheng, Zhiwei Chen, Huimin Shen, Fei Li

**Affiliations:** 1 Center of Hematology, The First Affiliated Hospital of Nanchang University, Nanchang, China; 2 Jiangxi Clinical Research Center for Hematologic Disease, Nanchang, China; 3 Institute of Lymphoma and Myeloma, Nanchang University, Nanchang, China

**Keywords:** Hemophagocytic lymphohistiocytosis, Coagulation index, Clinical features, Prognosis

## Abstract

Secondary hemophagocytic lymphohistiocytosis (HLH) is a life-threatening disease. In the present retrospective study, we aimed to investigate coagulation disorders and their outcome implications in patients with secondary HLH. We evaluated clinical characteristics and the relationship between coagulation indices and prognosis in HLH patients (*n* = 141). The information, including clinical symptoms, laboratory indicators, and coagulation indices, was evaluated. Coagulation disorders and bleeding events occurred in 95 (67.4%) and 60 (42.6%) patients, respectively. A coagulation index analysis primarily showed elevated levels of D-Dimer, the international standardized ratio (INR), prothrombin time (PT), activated partial thromboplastin time (APTT), and thrombin time (TT), while the prothrombin activity, fibrinogen levels, and platelet levels were significantly decreased. Dominant disseminated intravascular coagulation (DIC) occurred in 76 patients (53.9%). Patients with lymphoma-associated hemophagocytic syndrome (LAHS) frequently exhibited apparent coagulation disorders. Multivariate analysis revealed that age ≥ 29.5 years, bleeding events, APTT ≥ 47.3 s, fibrinogen ≤ 1.68 g/L, and absolute neutrophil counts (ANC) of ≤ 1.21 × 10^9^/L were independent prognostic factors. We thereby devised a prognostic scoring system and stratified patients into low-risk (0–2 points), intermediate-risk (3–4 points), and high-risk (5–7 points) groups, and the 1-year overall survival rates in the above-mentioned groups were 66.40%, 40.00%, and 2.30%, respectively (*P* < 0.0001). In conclusion, coagulation dysfunctions and bleeding tendencies were common characteristics in HLH patients. We constructed a novel prognostic score model based on APTT, fibrinogen level, ANC, age, and bleeding events, which had superior prognostic value compared with these markers alone.

**Supplementary information:**

The online version contains supplementary material available at 10.1007/s00277-023-05398-w.

## Introduction

Hemophagocytic lymphohistiocytosis (HLH), also known as hemophagocytic syndrome (HPS), is a life-threatening immune system disorder characterized by cytokine storms and overwhelming inflammation [1]. Cytotoxic T cells and macrophages cause multi-organ damage, hemophagocytosis, and severe systemic inflammation [2–4]. HLH patients present with a wide spectrum of clinical manifestations, including fever, cytopenia, hepatosplenomegaly, abnormal liver function, and coagulopathies.

Coagulopathies, which can occur throughout the disease course, are major factors responsible for patient mortality. Approximately 68% of critically ill HLH patients present with coagulopathies during the course of the disease [5], and abnormal coagulation parameters (especially decreased fibrinogen (FIB) levels) are common in these patients [6, 7]. Some patients develop bleeding in the skin, mucous membranes, and even major organs, such as life-threatening intracranial, gastrointestinal tract, and pulmonary hemorrhages. Disseminated intravascular coagulation (DIC) may also occur in HLH patients [8, 9]. DIC and thrombocytopenia are reported to be associated with adverse outcomes in HLH patients [6, 7]. Some retrospective studies have indicated that low FIB levels appear to be highly correlated with poor prognoses [6, 10]. However, only sparse data are available regarding critically ill HLH patients presenting with coagulation disorders. The recognition and understanding of the clinical characteristics of coagulopathy and its prognostic effects in HLH patients are currently limited.

In this retrospective study, we aimed to describe the characteristics of coagulopathies and coagulopathy-associated injuries in 141 HLH patients. We also assessed whether coagulation abnormalities and bleeding events influenced disease outcomes. Moreover, we constructed a novel and widely applicable prognostic scoring system based on coagulation parameters.

## Methods

### Subjects and evaluations

From November 2013 to May 2020, a total of 141 patients with secondary HLH were admitted to the First Affiliated Hospital of Nanchang University (Nanchang, China). The patients’ medical information, including clinical manifestations, underlying diseases, laboratory findings, treatments, and outcomes, was reviewed. Laboratory indices included the peripheral blood examinations, such as full blood count, alanine aminotransferase (ALT), aspartate aminotransferase (AST), albumin, bilirubin (BIL), triglyceride, lactate dehydrogenase (LDH), and D-Dimer (D-D) levels, prothrombin time (PT), activated partial thromboplastin time (APTT), the international normalized ratio (INR), prothrombin time activity (PTA), thrombin time (TT), FIB and serum ferritin levels, blood immunology, virology, bacteriology, bone marrow cytology, immunotyping by flow cytometry, bone marrow histology and immunohistochemistry, and imaging tests for the liver, spleen, and lymph nodes (including B-scan ultrasonography examination and computed tomography). Epstein-Barr virus (EBV) and cytomegalovirus (CMV) infections were diagnosed based on the detection of immunoglobulin M (IgM) antibodies or high levels of EBV or CMV DNA. Moreover, data regarding bleeding events during the course of the disease were also collected.

### Diagnostic criteria

All patients were diagnosed according to the revised diagnostic criteria guidelines within the definitive HLH-2004 protocol [11]. The diagnostic criteria for DIC adopts the ISTH overt DIC diagnostic scoring system is according to the parameters as follows: (1) PLT > 100 × 10^9^/L is 0 point; 50 × 10^9^/L < PLT ≤ 100 × 10^9^/L is 1 point, PLT < 50 × 10^9^/L is 2 points; (2) No increase in the level of fibrin degradation products or D-Dimer is 0 points, moderate increase is 2 points, significant increase is 3 points (D-Dimer < 1 mg/L is not increased, D-Dimer ≥ 1 mg/L and < 3 mg/L is moderately increased, and D-Dimer ≥ 3 mg/L is significantly increased); (3) PT < 3 s is 0 point, 3 s ≤ PT < 6 s is 1 point, PT ≥ 6 s is 2 points; (4) FIB level > 1.00 g/L is 0 point, FIB ≤ 1.00 g/L is 1 point. The total ISTH score ≥ 5 points was diagnosed as overt DIC, and the score < 5 points was defined as non-overt DIC.

### Treatment regimens

Of the 141 evaluated HLH patients, 85 patients received the following treatment regimens: 1) the HLH-94/04 protocol in 25 cases (dexamethasone and etoposide), 2) the DEP protocol in 33 cases (liposomal doxorubicin, etoposide, and methylprednisolone), and 3) immunotherapy in 27 cases (corticosteroids or intravenous immunoglobulin). Moreover, 26 patients only received supportive and symptomatic treatments but no HLH treatments due to early death. Supportive and symptomatic treatments included antibiotics, antivirals, drugs for liver dysfunctions, transfusion of blood components, such as red cells, FIB, or fresh frozen plasma. Besides, 22 patients abandoned HLH treatments and were discharged from the hospital. Eight patients received mixed treatments, such as other chemotherapy or immunotherapies.

The drug regimens were as follows: HLH-94 regimen, dexamethasone at 10 mg/m^2^/day during weeks 1 and 2, 5 mg/m^2^/day during weeks 3 and 4, 2.5 mg/m^2^/day during weeks 5 and 6, and 1.25 mg/m^2^/day during weeks 7 and 8; etoposide at 100 mg/m^2^/day two times weekly during weeks 1 and 2 and 100 mg/m^2^/day once each week during weeks 3–8. DEP regimen, liposomal doxorubicin (doxorubicin hydrochloride liposome injection) 25 mg/m^2^/day, day 1; etoposide at 100 mg/m^2^/day on the first day of every week; and methylprednisolone at 15 mg/kg/day on days 1 to 3, 0.75 mg/kg/day on days 4 to 7, and 0.25 mg/kg/day on days 8 to 10.

### Statistical analysis

Statistical analysis was performed using SPSS 22.0 (Chicago, IL, USA) and GraphPad Prism 7.0 (San Diego, CA, USA) statistical software. T-tests were used for comparisons of normally distributed measurement data, while non-parametric tests were used for comparisons of data with non-normal distributions, and chi-square test was used for comparison of categorical data. Receiver operating characteristic (ROC) curve analysis was conducted to determine the optimal cut-off values for various clinical indicators. The Kaplan–Meier method was used for survival analysis, and the log-rank test was conducted for intergroup comparisons. Overall survival (OS) was defined as the time from the date of diagnosis to the date of death of any triggering event. A Cox proportional hazard regression model was used for multivariate analysis. The hazard ratios (HRs) of the independent risk factors, determined by Cox multivariate analysis, were compared. The score of the influencing factor corresponding to the smallest HR was set to 1, and the other HRs were divided by the smallest regression coefficient, followed by a rounding off of the quotients. The scores corresponding to different influencing factors were calculated. Finally, the patients were stratified into different groups based on the results of the analysis. *P*-values of < 0.05 were considered statistically significant.

## Results

### Overviews of patients

A total of 141 HLH patients (82 males and 59 females) were included in this study. The median age was 47 years (range, 9–89 years). Two patients were under 10 years of age. None of the patients fulfilled the diagnostic criteria for primary HLH. In terms of underlying diseases, there were 47 infection-associated HLH cases, including 33 patients with EBV infection, one patient with histoplasmosis, and 13 patients with a bacterial infection. Moreover, 72 cases were malignancy-associated HLH, including 68 patients with lymphoma, one patient with a solid tumor, and three patients with aggressive natural killer (NK) cell leukemia. In addition, there were eight patients with macrophage activation syndrome, including four patients with systemic lupus erythematosus, one patient with Sjogren’s syndrome, and three patients with unclassified connective tissue disease. Specific causes of HLH could not be identified in the remaining 14 patients.

### Bleeding sites and coagulopathy disorders

In the present study, 60 (42.6%) of the 141 HLH patients presented with bleeding of varying degrees, including skin petechiae (*n* = 28), gastrointestinal bleeding (*n* = 25), respiratory tract bleeding (*n* = 10), epistaxis (*n* = 10), gingival bleeding (*n* = 4), hematuria (*n* = 3), eye bleeding (*n* = 3), and/or intracranial hemorrhage (*n* = 3). Other patients exhibited oral bleeding (*n* = 2), intra-abdominal hemorrhage (*n* = 1), and bleeding at the biopsy site (*n* = 1) (Table 1). The incidence of bleeding events was not associated with sex, age, hemophagocytosis in the bone marrow, or lymphoma-associated HLH (*P* > 0.05). Most patients had one bleeding site (*n* = 41), while twelve, five, and two patients had two, three, and ≥ three hemorrhage sites, respectively. Various abnormalities of coagulopathy indicators were described in Table 1.

**Table 1:** Clinical bleeding site and laboratory characteristics in 141 HLH patients

Clinical characteristics	Cases (%)	Laboratory characteristics	Cases (%)
Hemorrhage	60 (42.6%)	ANC < 1 × 10^9^/L	56 (39.7%)
Petechiae	28 (19.9%)	Hemoglobin < 90 g/L	75 (53.2%)
Gastrointestinal tract bleeding	25 (17.7%)	Platelet < 100 × 10^9^/L	111 (78.7%)
Respiratory tract bleeding	10 (7.10%)	D-Dimer > 0.5 mg/L	136 (96.5%)
Epistaxis	10 (7.10%)	Prolonged PT (S)	34 (24.1%)
Bleeding gums	4 (2.84%)	Prolonged APTT (S)	75 (53.2%)
Hematuria	3 (2.13%)	INR > 1.2	62 (45.3%)
Eye bleeding	3 (2.13%)	PTA < 80%	90 (65.2%)
Intracranial hemorrhage	3 (2.13%)	Prolonged TT (S)	41 (29.9%)
Oral bleeding	2 (1.42%)	FIB ≤ 1.50 g/L	64 (45.4%)
Intraabdominal hemorrhage	1 (0.71%)		
Bleeding at the biopsy site	1 (0.71%)		
Number of the bleeding site			
One	41 (29.1%)		
Two	12 (8.51%)		
Three	5 (3.55%)		
More than three	2 (1.42%)		

ANC: absolute neutrophil count; PT: prothrombin time. KPTT: kaolin partial thromboplastin time. TT: Thrombin time. Prolonged PT was defined as more than 3 S compared to the normal control. Prolonged KPTT was defined as more than 10 S compared to the normal control. Prolonged TT was defined as more than 3 S compared to the normal control. INR: international normalized ratio. PTA: prothrombin time activity. FIB: fibrinogen

### DIC in HLH patients

We subsequently evaluated the characteristics of DIC in this group. The median international society on thrombosis and haemostasis (ISTH) score at the time of admission was 5 (range: 0–8). Based on their initial ISTH score, patients were dichotomized into ISTH ≥ 5 (*n* = 76, 53.9%) and ISTH < 5 (*n* = 65, 46.1%) groups (Table 2). It was worth noting that the proportion of patients with lymphoma in the ISTH ≥ 5 group was significantly higher compared with the ISTH < 5 group (57.9% versus 36.9%, *P* = 0.013) (Table 2). Moreover, PT, D-D, APTT, TT, and INR values were significantly elevated or prolonged, while FIB and PTA values were significantly reduced in the ISTH ≥ 5 group (all *P* < 0.05). Moreover, the incidence of bleeding events in the ISTH ≥ 5 group was higher compared with the ISTH < 5 group (53.9% versus 29.2%, *P* = 0.003).

**Table 2:** The coagulation disorders between the ISTH DIC score ≥ 5 and ISTH DIC score < 5 groups

Parameter	ISTH DIC score ≥ 5 group (*n* = 76)	ISTH DIC score < 5 group (*n* = 65)	*P*-value
Age [years, M (range)]	48.0 (9.0–89.0)	47.0 (10.0–80.0)	0.448
Sex (cases, M/F)	48/28	34/31	0.193
Platelet [× 10^9^/L, M (range)]	27(5.00–268.00)	85(4.00–317.00)	< 0.001
PT [s, M (range)]	15.15 (9.70- 26.00)	12.60 (9.70–16.20)	< 0.001
PTA [%, M (range)]	63.30 (23.40–163.70)	76.55 (38.30–142.90)	< 0.001
INR [M (range)]	1.27 (0.81–2.31)	1.13 (0.86–1.46)	< 0.001
APTT [s, M (range)]	46.30 (20.10–117.10)	37.20 (23.70–68.90)	< 0.001
FIB [g/L, M (range)]	1.28 (0.49–8.35)	2.60 (0.87–7.62)	< 0.001
TT [s, M (range)]	20.05 (13.10–62.20)	18.00 (13.40–37.30)	0.014
D-Dimer (mg/L)	7.37 (0.05–203.68)	2.80 (0.19–56.37)	< 0.001
Hemorrhagic events (cases, Y/N)	41/35	19/46	0.003
LAHS (cases, Y/N)	44/32	24/41	0.013

FIB: fibrinogen. LAHS: lymphoma-associated hemophagocytic syndrome

### Coagulation disorders in patients with lymphoma and EBV-associated HLH

There were more lymphoma patients in the ISTH ≥ 5 group, suggesting that lymphoma patients were likely to present with more severe coagulation disorders. Therefore, we compared coagulation indicators between the lymphoma-associated hemophagocytic syndrome (LAHS) group (*n* = 68) and non-LAHS group (*n* = 73). PT was significantly prolonged in the LAHS group compared with the non-LAHS group (14.1 s (range: 9.70–25.70 s) versus 13.1 s (range: 9.70–26.00 s), *P* = 0.03) (Supple. Table 1). Meanwhile, EBV-DNA-positive patients showed a significantly prolonged APTT (range: 21.70–117.10 s, *P* = 0.003) and lower levels of FIB (range: 0.49–8.35 g/L, *P* = 0.024) compared with EBV-negative patients (Supple. Table 2). In addition, the APTT was also prolonged in the EBV-positive LAHS group (range: 21.70–75.80 s, *P* = 0.015) (Supple. Table 3). These results indicated that more coagulation disorders occurred in LAHS and EBV-associated HLH, predicting the importance and potential prognostic values of these indicators.

### Survival analysis

**Fig. 1:**
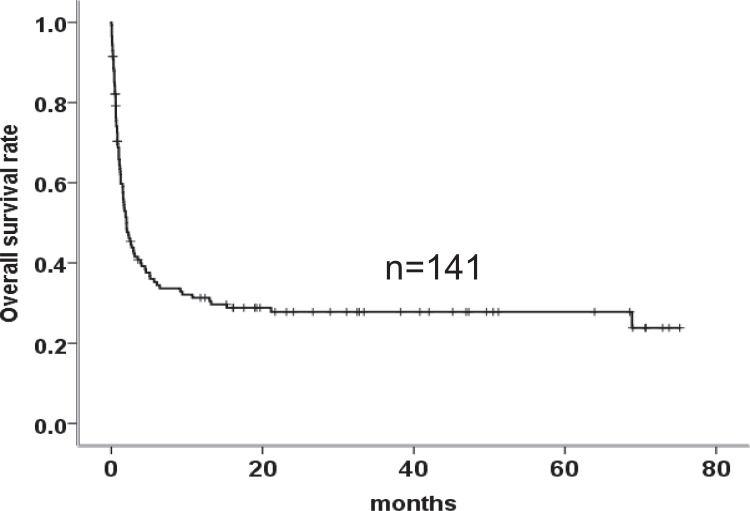
Survival outcomes in 141 HLH patients

**Fig. 2:**
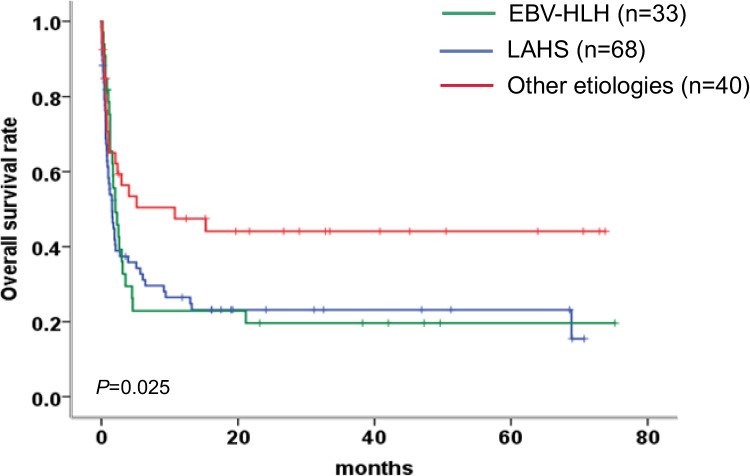
EBV-HLH and LAHS patients had significantly worse OS compared with those patients without EBV infection and lymphoma (*P* = 0.025)

The median follow-up time was 32.8 months (range 0.03–75.2 months), and 97/141 patients (68.8%) died. The median OS of the entire cohort was 2.0 months, and the 1-year OS was 31.3%. Figure 1 shows the survival curve of the 141 enrolled HLH patients, and 47 patients died in the first 30 days (33.3%). In addition, EBV-HLH and LAHS patients had significantly poor OS compared with those patients without EBV infection and lymphoma (2.03 versus 1.53 versus 10.7 months; *P* = 0.025) (Fig. 2).

To further analyze the potential risk factors affecting HLH prognosis, we compared routine laboratory parameters between patients in the dead and survived groups listed in Table 3. Optimal cut-off values, including those for APTT, FIB, and ANC, were determined according to ROC and AUC analyses. We found that age, sex, APTT values, FIB levels, white blood cell counts, hemoglobin levels, platelet counts, absolute neutrophil counts (ANC), ISTH scores, and hemorrhagic events were associated with an increased risk of death. Next, we conducted Cox multivariate regression and identified that age ≥ 29.5 years (HR = 3.319, 95% confidence interval [CI]: 1.766–6.239, *P* < 0.001), bleeding events (HR = 3.685, 95% CI: 2.331–5.825, *P* < 0.001), APTT ≥ 47.3 s (HR = 1.684, 95% CI: 1.058–2.681, *P* = 0.028), FIB ≤ 1.68 g/L (HR = 1.742, 95% CI: 1.108–2.741, *P* = 0.016), and ANC ≤ 1.21 × 10^9^/L (HR = 1.671, 95% CI: 1.059–2.635, *P* = 0.027) were independent risk factors for poor OS (Table 4).

**Table 3:** The characteristics of clinical parameters between the non-survival and survival groups

Parameter	Non-survival group (*n* = 97)	Survival group (*n* = 44)	*P*-value
Age [years, M (range)]	51.0 (10.0–89.0)	37.0 (9.0–76.0)	< 0.001
Sex (cases, M/F)	63/34	19/25	0.015
PT [s, M (range)]	13.80 (9.70- 26.00)	13.05 (10.00–19.80)	0.094
PTA [%, M (range)]	69.45 (23.40–163.70)	75.15 (32.90–142.90)	0.174
INR [M (range)]	1.19 (0.81–2.31)	1.16 (0.86–1.77)	0.390
APTT [s, M (range)]	44.30 (21.70–117.10)	38.95 (20.10–68.90)	0.022
FIB [g/L, M (range)]	1.44 (0.49–8.35)	2.53 (0.53–7.62)	0.001
TT [s, M (range)]	19.90 (13.10–62.20)	17.80 (13.40–37.30)	0.076
D-Dimer (mg/L)	5.30 (0.05–203.68)	6.06 (0.25–131.50)	0.940
WBC [× 10^9^/L, M (range)]	1.82 (0.04–15.99)	3.56 (0.25–25.19)	< 0.001
Hemoglobin [g/L, M (range)]	84.00 (37.00–152.00)	94.50 (53.00–147.00)	0.014
Platelet [× 10^9^/L, M (range)]	42.00 (4.00–290.00)	79.00 (5.00–317.00)	0.004
ANC [× 10^9^/L, M (range)]	0.98 (0–13.77)	2.04 (0.03–17.38)	< 0.001
Hemorrhagic events (cases, Y/N)	51/46	9/35	< 0.001
Hemophagocytosis* (cases, Y/N)	60/32	29/13	0.663
LAHS (cases, Y/N)	52/45	16/28	0.058
ISTH score ≥ 5 (cases, Y/N)	60/37	16/28	0.005
ISTH score [M (range)]	5 (0–8)	4 (0–8)	0.005

^*^Presence of hemophagocytosis was assessed in 92 patients in the non-survival group and 42 patients in the survival group

**Table 4:** Cox multivariate analysis of prognostic factors in HLH patients

Risk factor	HR	95% confidence interval	*P*-value
Age ≥ 29.5 years	3.319	1.766–6.239	0.000
Occurrence of hemorrhagic event	3.685	2.331–5.825	0.000
APTT ≥ 47.3 s	1.684	1.058–2.681	0.028
FIB ≤ 1.68 g/L	1.742	1.108–2.741	0.016
ANC ≤ 1.21 × 10^9^/L	1.671	1.059–2.635	0.027

Consistent with the above-mentioned results, patients with the FIB ≤ 1.68 g/L had lower platelet counts, and more bleeding events than those with the FIB > 1.68 g/L (*P* < 0.05). In addition, these patients had higher levels of AST and LDH and lower levels of serum albumin (*P* < 0.05). This finding showed that persistent hypofibrinemia was closely associated with poor prognoses.

Given that 33.3% (*n* = 47) of patients died within 30 days, we further analyzed the factors associated with early death. As we thought, patients with early death had significantly worse indicators, such as age, PT, APTT, FIB, TT, white blood cell count, ANC, and albumin values (all *P* < 0.05) (Table S4). Even more, patients with initial ISTH scores of ≥ 5 had a higher 30-day death rate (70.2% versus 45.7%, *P* = 0.006), with the incidence of bleeding events being 68.1% (versus 29.8%, *P* < 0.001).

### Prognosis-prediction model

**Fig. 3:**
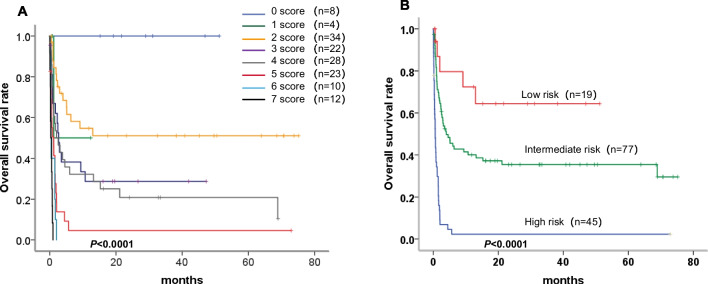
Kaplan–Meier survival curves showing the OS according to the prognostic scores. **A** The patients were scored from 0 to 7 points, divided into eight groups according to the prognostic score. Survival rates deteriorated with the increase in the prognostic scores (*P* < 0.0001). **B** The survival time of patients was divided into the low-risk (0–2 points), intermediate-risk (3–4 points), and high-risk groups (5–7 points) according to prognostic scores from the novel model (*P* < 0.0001)

As mentioned above, age, bleeding events, APTT ≥ 47.3 s, FIB ≤ 1.68 g/L, and ANC ≤ 1.21 × 10^9^/L were independent risk factors for poor OS. We found the following optimal cut-off as well as sensitivity and specificity values: APTT, 47.3 s, 0.423, 0.795; FIB, 1.68 g/L, 0.619, 0.750; and ANC, 1.21 × 10^9^/L, 0.546, 0.818, respectively. We constructed a prognosis-prediction model (score range, 0–7) from Fig. 3A, showing that the survival rate deteriorated with the increase of prognostic scores (*P* < 0.0001). According to the associated HR in the Cox model, age ≥ 29.5 years and bleeding events were counted as two points, and APTT ≥ 47.3 s, FIB ≤ 1.68 g/L, and ANC ≤ 1.21 × 10^9^/L were each counted as one point. The patients were further categorized based on the total prognostic score into low-risk (0–2 points), intermediate-risk (3–4 points), and high-risk groups (5–7 points) (Table 5). Finally, Kaplan–Meier survival curves are shown in Fig. 3B. Our prognostic score based on coagulation indices clearly classified the enrolled patients into different survival groups. The 1-year OS values in the low-risk, intermediate-risk, and high-risk patients were 66.40%, 40.00%, and 2.30%, respectively (*P* < 0.0001).

**Table 5:** HLH patients was classified different risk subgroups according to different prognostic scores

Total prognostic score	Prognosis group	Cases	Percentage	1-year OS
0–2	Low-risk	19	13.50%	66.40%
3–4	Moderate-risk	77	54.60%	40.00%
5–7	High-risk	45	31.90%	2.30%

OS: overall survival

## Discussion

To the best of our knowledge, few studies have reported on coagulation disorders in HLH patients, though adverse outcomes in these patients have been reported previously (i.e., bleeding complications). Valade et al. [12] have reported a median bleeding event duration of 3.5 days since initial diagnosis in critically ill HLH patients, with a frequency of approximately 20%. Jin et al. [13] have retrospectively evaluated 52 HLH patients with gastrointestinal bleeding, reporting that 90% of the patients have coagulopathies, and 69% of the patients have concomitant bleeding from other sites (such as petechiae, intracranial hemorrhage, and oral hemorrhage). Guo et al. [14] have found that the platelet counts of HLH patients with bleeding complications are significantly decreased, and 60% of these patients also have decreased FIB levels, indicating that hemorrhagic diathesis can indirectly reflect impaired coagulation. In our present study, bleeding was a common clinical manifestation that could occur in the early disease stage or throughout the disease course in HLH patients. Common bleeding sites included the skin, gastrointestinal tract, respiratory tract, nasal cavity, and gingivae.

Numerous studies have indicated that some critically ill HLH patients have fatal hemorrhage that is closely associated with DIC. Merrill et al. [15] have conducted a retrospective study, showing that 41.3% of HLH patients have a major hemorrhage, and 83.3% of patients with hemorrhage present with DIC. A French study consisting of 117 HLH patients has indicated that 26 (22%) patients present with severe bleeding complications, including five patients who die of hemorrhagic shock. DIC is found in half of the patients with a higher mortality rate [12]. Correspondingly, our data showed that HLH patients with concomitant thrombocytopenia, coagulopathy, or DIC had a higher risk of bleeding. Moreover, patients with ISTH scores of ≥ 5 had a higher incidence of bleeding and mortality. Hence, patients with overt DIC typically exhibit severe hemorrhagic diathesis and poor prognoses, and such patients must be closely monitored.

The primary coagulation abnormalities seen in this study were thrombocytopenia, prolonged APTT, elevated INR and D-D values, and decreased FIB levels. Thrombocytopenia may occur due to severe cytokine storms interfering with bone marrow hematopoiesis, the destruction of hematopoietic precursor cells by cytokines, and platelet destruction by over-activated macrophages [16–18]. The decrease of FIB level is related to the increase of consumption and reduction of synthesis caused by various reasons. Severe cytokine storms can damage vascular endothelial cells, induce tissue factor release and collagen exposure, and initiate intrinsic and extrinsic coagulation pathways (both of which consume FIB) [17, 19]. Moreover, abnormally activated macrophages can up-regulate the urokinase plasminogen activator surface receptor on monocytes via the action of interleukin 6, tumor necrosis factor-α, and other cytokines, in addition to binding to the urokinase-type plasminogen activator, thereby increasing the local fibrinolytic function of cells and ultimately leading to FIB degradation [20]. Patients with impaired liver function have a reduced ability to synthesize FIB and other related coagulation factors, which can also lead to the imbalance of fibrinolytic pathway. A retrospective study by Tang et al. [21] has found that prolonged PT and APTT occur in 67.39% and 76.09% of HLH patients with abnormal liver function, respectively. Therefore, it is evident that activation of severe cytokine storms, impaired coagulation function, and severe hemorrhagic diathesis promote disease progression and may lead to DIC or multiple organ dysfunction.

The 1-year OS of all patients was 31.3%, which was similar to the OS reported in a previous investigation (35.2%) [13]. Our data also showed that age ≥ 29.5 years, APTT ≥ 47.3 s, FIB ≤ 1.68 g/L, ANC ≤ 1.21 × 10^9^/L, and bleeding events were independent mortality risk factors, consistent with the results of a study enrolling 171 HLH patients [22]. Another retrospective study has shown that 11.3% and 28.8% of HLH patients die within 7 and 30 days after admission, respectively, proposing that prolonged APTT (> 48 s), prolonged PT (> 14 s), elevated INR (> 1.5), and decreased FIB (≤ 1.50 g/L) values are strong risk factors for early mortality [23]. In our cohort, patients with a FIB level of ≤ 1.68 g/L had more prominent prolongation of PT and APTT, lower platelet counts, more bleeding events, higher levels of AST and LDH and lower levels of serum albumin. Yin et al. [24] have found that FIB levels show a non-linear negative correlation with mortality in HLH patients. When FIB levels were ≤ 1.76 g/L, survival was decreased with a reduction in FIB levels. Several studies support the assertion that FIB levels are associated with the occurrence of severe hemorrhage as well as with disease severity and early mortality, with various cut-off values between 1.5 and 2 g/L. Differences in cut-off values may account for the considerable differences in case counts across studies. These results indicate that hypofibrinemia exists throughout the course of the disease and is an important indicator of poor prognoses.

Discerning patients in danger of poor prognoses is crucial in the treatment of HLH. Pan et al. [8] have developed a prognostic risk score for pediatric patients with non-malignancy-associated secondary HLH, defined by the following parameters measured at diagnosis: hemoglobin < 60 g/L, platelets < 30 × 10^9^/L, and albumin < 25 g/L. This risk score may identify patients at high risk of disease progression. Moreover, in adult patients with newly diagnosed HLH, Zhao et al. [22] have found that older patients (≥ 54 years) as well as those with thrombocytopenia (< 39.5 × 10^9^/L), prolonged APTT (≥ 54 s), hypertriglyceridemia (≥ 3.23 mmol/L), elevated LDH (≥ 1,300 U/L), and malignancy are at higher risk of early death. Additionally, these researchers have established a prognostic model, which can classify HLH patients into four groups according to the number of risk factors present at the time of diagnosis. However, there is no specific prognostic score for HLH patients that takes into account different coagulation indices, and prognostic models for risk stratification are not completely elucidated. According to the results of the Cox regression in this cohort, we established a novel prognosis-prediction model based on coagulation indicators, including age, APTT, FIB, ANC, and bleeding events. Using this model, we stratified patients into three obviously different survival populations. The 1-year OS rates in the low-risk, intermediate-risk, and high-risk groups were 66.40%, 40.00%, and 2.30%, respectively. Constructing an applicable clinical model is important in terms of recognizing and identifying critically ill patients at high risk of early death.

In summary, our study reported the characteristics of bleeding sites and coagulation disorders in HLH patients and showed that abnormalities in coagulation indicators were closely associated with a high risk of mortality in HLH patients. Moreover, we constructed a novel and widely applicable clinical model for performing risk stratifications with regard to data on age, APTT, FIB, ANC, and bleeding events. However, this study had several limitations due to its retrospective and single-center design. We believed that our results provided an awareness of the importance of coagulation disorders in HLH patients, which could help clinicians recognize critically ill patients early and guide timely interventions, thereby reducing the high mortality rate.

### Supplementary information

Below is the link to the electronic supplementary material.

Supplementary file1 (DOCX 24 KB)

## Data Availability

The data used for this study, though not available in a public repository, will be made available to other researchers upon reasonable request.
