# Supplementary material for: A novel prognosis-prediction model based on coagulation indicators in secondary hemophagocytic lymphohistiocytosis
Source: Ann Hematol. 2023 Aug 10;102(11):3251–9. doi: 10.1007/s00277-023-05398-w (PMC10567857; doi:10.1007/s00277-023-05398-w)
Supplement: Supplementary file 1 — Supplementary file1 (DOCX 24 KB) [file 277_2023_5398_MOESM1_ESM.docx]

**Supplementary material**

Table S1. The coagulation disorders between the LAHS and non-LAHS groups.

| Parameter | LAHS group  (n = 68) | Non-LAHS group  (n = 73) | *P*-value |
| --- | --- | --- | --- |
| PT [s, M (range) ] | 14.10 (9.70-25.70) | 13.10 (9.70-26.00) | 0.030 |
| PTA [%, M (range) ] | 68.00 (32.30-119.50) | 74.25 (23.40-163.70) | 0.071 |
| INR [M (range) ] | 1.22 (0.90-1.82) | 1.17 (0.81-2.31) | 0.083 |
| APTT [s, M (range) ] | 43.90 (20.10-116.30) | 40.00 (23.70-117.10) | 0.402 |
| FIB [g/L, M (range) ] | 1.45(0.49-8.35) | 1.90 (0.50-7.13) | 0.310 |
| TT [s, M (range) ] | 19.40 (13.10-37.00) | 19.75 (13.40-62.20) | 0.750 |
| D-Dimer (mg/L) | 5.50 (0.73-203.68) | 6.10 (0.05-131.50) | 0.755 |
| Hemorrhagic events (cases, Y/N) | 31/37 | 29/44 | 0.482 |
| ISTH score ≥ 5 (cases, Y/N) | 44/24 | 32/41 | 0.013 |
| ISTH score [M (range) ] | 5 (1-8) | 4 (0-8) | 0.027 |

LAHS: lymphoma-associated hemophagocytic syndrome. PT: prothrombin time. PTA: prothrombin activity. INR: international normalized ratio. KPTT: kaolin partial thromboplastin time. FIB: fibrinogen. TT: Thrombin time. ISTH: International Society on Thrombosis and Haemostasis.

Table S2. The coagulation disorders between the EBV positive and EBV negative groups.

| Parameter | EBV positive group  (n = 76) | EBV negative group  (n = 59) | *P*-value |
| --- | --- | --- | --- |
| PT [s, M (range) ] | 13.65 (9.70-26.00) | 13.40 (10.00-24.20) | 0.260 |
| PTA [%, M (range) ] | 71.50 (23.4-163.70) | 71.90 (32.20-142.90) | 0.607 |
| INR [M (range) ] | 1.18 (0.81-2.31) | 1.19 (0.86-2.22) | 0.960 |
| APTT [s, M (range) ] | 44.95 (21.70-117.10) | 37.20 (20.10-116.30) | 0.003 |
| FIB [g/L, M (range) ] | 1.48(0.49-8.35) | 2.33 (0.60-7.13) | 0.024 |
| TT [s, M (range) ] | 19.90 (13.10-62.20) | 17.80 (13.40-37.30) | 0.114 |
| D-Dimer (mg/L) | 5.72 (0.05-71.01) | 6.02(0.19-203.68) | 0.952 |
| Hemorrhagic events (cases, Y/N) | 31/45 | 24/35 | 0.990 |
| ISTH score ≥ 5 (cases, Y/N) | 45/31 | 27/32 | 0.120 |
| ISTH score [M (range) ] | 5 (0-8) | 4 (0-8) | 0.059 |

Table S3. The coagulation disorders between the EBV positive or negative LAHS patients.

| Parameter | LAHS with EBV positive (n = 36) | LAHS with EBV negative  (n =29) | *P*-value |
| --- | --- | --- | --- |
| PT [s, M (range) ] | 14.75(9.70-25.70) | 13.60(11.10-19.80) | 0.180 |
| PTA [%, M (range) ] | 68.40(37.20-119.50) | 71.90 (32.30-104.70) | 0.839 |
| INR [M (range) ] | 1.21(0.90-1.82) | 1.20 (0.99-1.77) | 0.917 |
| APTT [s, M (range) ] | 46.95 (21.70-75.80) | 37.60 (20.10-116.30) | 0.015 |
| FIB [g/L, M (range) ] | 1.41(0.49-8.35) | 2.08 (0.60-6.97) | 0.243 |
| TT [s, M (range) ] | 19.70 (13.10-35.00) | 17.60 (14.10-37.00) | 0.390 |
| D-Dimer (mg/L) | 4.48 (0.73-41.89) | 6.22 (1.44-203.68) | 0.143 |
| Hemorrhagic events (cases, Y/N) | 15/21 | 13/16 | 0.798 |
| ISTH score ≥ 5 (cases, Y/N) | 24/12 | 17/12 | 0.504 |
| ISTH score [M (range) ] | 6 (1-8) | 5 (2-8) | 0.163 |

Table S4. The comparison of clinical parameters between the early death group and survival groups.

| Parameter | Die within 30 days  (n=47) | Survival within 30 days  (n=94) | *P*-value |
| --- | --- | --- | --- |
| Age [years, M (range) ] | 53.0 (13.0-89.0) | 46.5 (9.0-76.0) | \| 0.004 0.004 \| \| --- \| |
| Sex (cases, M/F) | 28/19 | 54/40 | 0.809 |
| PT [s, M (range) ] | 15.10 (10.10-26.00) | 13.10 (9.70-19.80) | 0.001 |
| PTA [%, M (range) ] | 64.75 (23.40-163.70) | 73.35 (32.90-142.90) | 0.066 |
| INR [M (range) ] | 1.22 (0.81-2.31) | 1.17 (0.86-1.77) | 0.092 |
| APTT [s, M (range) ] | 47.80 (28.90-117.10) | 40.15 (20.10-94.80) | 0.001 |
| FIB [g/L, M (range) ] | 1.19 (0.49-8.35）6 | 2.09 (0.53-7.62) | 0.001 |
| TT [s, M (range) ] | 20.00 (13.60-62.20) | 18.80 (13.10-37.30) | 0.041 |
| D-Dimer (mg/L) | 5.86 (0.38-71.01) | 4.75 (0.05-203.68) | 0.153 |
| WBC [×10^9^/L, M (range) ] | 1.66 (0.04-15.99) | 2.60 (0.18-25.19) | 0.016 |
| Hemoglobin [g/L,M(range) ] | 87.00 (37.00-136.00) | 88.00 (52.00-152.00) | 0.326 |
| Platelet [×10^9^/L, M (range) ] | 39.00 (6.00-290.00) | 50.00 (4.00-317.00) | 0.208 |
| ANC [×10^9^/L, M (range) ] | 0.90 (0-13.77) | 1.53 (0.-17.38) | \| 0.016 \| \| --- \| |
| ALT [U/L, M (range) ] | 63.00 (9.00-646.00） | 51.50 (6.00-787.00) | 0.673 |
| AST [U/L, M (range) ] | 109.00 (16.00-1376.00) | 84.00 (8.00-3763.00) | 0.267 |
| DBIL [mmol/L, M (range) ] | 9.70 (2.50-273.00) | 6.60 (0.50-191.40) | 0.055 |
| Albumin [g/L, M (range) ] | 26.80 (16.70-35.40） | 28.20 (18.80-38.50) | 0.009 |
| LDH [U/L, M (range) ] | 826.00 (215.00-6013.00) | 720.00 (171.00-4589.00) | 0.173 |
| Triglyceride [mmol/L,M (range)] | 2.32 (0.36-6.24） | 2.30 (0.51-9.50) | 0.866 |
| Hemorrhagic events (cases, Y/N) | 32/15 | 28/66 | <0.001 |
| ISTH score ≥ 5 (cases, Y/N) | 33/14 | 43/51 | 0.006 |
| ISTH score [M (range) ] | 6 (2-8） | 4 (0-8） | <0.001 |
